# Early improvement in severely ill patients with pneumonia treated with ceftobiprole: a retrospective analysis of two major trials

**DOI:** 10.1186/s12879-019-3820-y

**Published:** 2019-02-26

**Authors:** Thomas W. L. Scheeren, Tobias Welte, Mikael Saulay, Marc Engelhardt, Anne Santerre-Henriksen, Kamal Hamed

**Affiliations:** 1 Department of Anesthesiology, University Medical Center Groningen, Hanzeplein 1, P.O. Box 30.001, 9700 RB Groningen, The Netherlands; 2 Department of Respiratory Medicine, Medizinische Hochschule Hannover, Carl-Neuberg-Straße1, 30625 Hannover, Germany; 3 ICON Clinical Research (Switzerland) GmbH, Gewerbestrasse 24, 4123 Allschwil, Switzerland; 4 Basilea Pharmaceutica International Ltd., Grenzacherstrasse 487, P.O. Box, 4005 Basel, Switzerland

**Keywords:** Ceftobiprole, Cephalosporin, Community-acquired pneumonia, Hospital-acquired pneumonia

## Abstract

**Background:**

Patients with pneumonia who are elderly or severely ill are at a particularly high risk of mortality. This post hoc retrospective analysis of data from two Phase III studies evaluated early improvement outcomes in subgroups of high-risk patients with community-acquired pneumonia (CAP) and hospital-acquired pneumonia (HAP, excluding ventilator-associated pneumonia [VAP]).

**Methods:**

One study included hospitalised CAP patients randomised to ceftobiprole or ceftriaxone ± linezolid treatment. The other study included HAP patients, who were randomised to ceftobiprole or ceftazidime plus linezolid treatment. The primary outcome was rate of early clinical response (Day 3 in CAP and Day 4 in HAP patients). Additional outcome measures included clinical cure at a test-of-cure visit, 30-day all-cause mortality and safety.

**Results:**

The overall high-risk group comprised 398 CAP patients and 307 HAP patients with risk factors present at baseline. The rate of early response was numerically higher in ceftobiprole-treated patients vs comparator-treated patients in the following high-risk groups: CAP patients aged ≥75 years (16.3% difference, 95% confidence interval [CI]: 1.8, 30.8); CAP patients with COPD (20.1% difference, 95% CI: 8.8, 31.1); all high-risk HAP patients (12.5% difference, 95% CI: 3.5, 21.4); HAP patients with >10 baseline comorbidities (15.3% difference, 95% CI: 0.3, 30.4).

**Conclusions:**

Previous studies show that ceftobiprole is an efficacious therapy for patients with pneumonia who are at high risk of poor outcomes. This post hoc analysis provides preliminary evidence that ceftobiprole treatment may have advantages over other antibiotics in terms of achieving early improvement in high-risk patients with HAP (excluding VAP) and in some subgroups of high-risk CAP patients.

**Trial registration:**

NCT00210964: registered September 21, 2005; NCT00229008: registered September 29, 2005; NCT00326287: registered May 16, 2006.

**Electronic supplementary material:**

The online version of this article (10.1186/s12879-019-3820-y) contains supplementary material, which is available to authorized users.

## Background

Pneumonia is a common bacterial infection, especially in the elderly. The elderly population is increasing worldwide [1] and consequently the clinical and economic burden of pneumonia is expected to increase in the future [2–4]. The severity and outcome of pneumonia is dependent on a variety of external factors, such as the causative pathogen and treatment approach [5]. Patient-related factors are also involved, with poorer outcomes in patients who are elderly (aged ≥65 years), immunocompromised, malnourished or with multiple comorbidities [5–8]. Elderly or severely ill patients with pneumonia often present with several of these factors, and are therefore at particularly high risk of mortality and morbidity [6, 9, 10].

The healthcare costs associated with pneumonia are high and represent a large economic burden [3, 11–16]. Furthermore, the costs associated with pneumonia increase substantially in elderly patients or those with a chronic or immunocompromising disease, because of a longer duration of inpatient hospitalisation and need for management of underlying comorbidities [11, 13, 17]. Clearly, it is therefore important to ensure that first-line treatment options for high-risk patients with pneumonia are effective in both improving patient outcomes and minimising the burden of pneumonia on the healthcare system.

Ceftobiprole medocaril is an advanced-generation intravenous (i.v.) cephalosporin antibiotic. It is the first cephalosporin approved in European countries for both community-acquired pneumonia (CAP) and hospital-acquired pneumonia (HAP) (excluding ventilator-associated pneumonia [VAP]) [18]. Ceftobiprole, the active moiety of ceftobiprole medocaril, has broad-spectrum bactericidal activity against a wide range of Gram-positive pathogens (including methicillin-resistant *Staphylococcus aureus* [MRSA] and penicillin- and ceftriaxone-resistant pneumococci) and Gram-negative pathogens (including *Enterobacteriaceae* strains not producing extended-spectrum β-lactamase and *Pseudomonas aeruginosa*) [18–20].

The safety and efficacy of ceftobiprole have been demonstrated in two large Phase III trials in patients with CAP and HAP. The first study was a double-blind, multicentre, randomised study in 638 hospitalised patients with CAP, which demonstrated that ceftobiprole was non-inferior to ceftriaxone ± linezolid [21]. The second study was a double-blind, multicentre, randomised study in 781 patients with HAP, which demonstrated that ceftobiprole treatment was non-inferior to a combined treatment including ceftazidime plus linezolid, although non-inferiority was not demonstrated in the subgroup of patients with VAP [22].

We performed a post hoc analysis of data from these two Phase III studies [21, 22] to evaluate early improvement outcomes in subgroups of high-risk patients treated with ceftobiprole, compared with the respective active-control therapies (ceftriaxone ± linezolid in CAP and ceftazidime plus linezolid in HAP). The post hoc analyses presented here include only patients with HAP, excluding VAP, in accordance with the approved indication for ceftobiprole [18]. All mentions of HAP patients hereafter exclude patients with VAP.

## Methods

### Study design

The study designs have previously been described in detail elsewhere [21, 22]. Briefly, the CAP study [ClinicalTrials.gov identifier NCT00326287] was a multicentre, international, double-blind, non-inferiority study of hospitalised adult patients with CAP undertaken in 103 centres between June 2006 and June 2007 [21]. Key inclusion criteria comprised a diagnosis of acute bacterial CAP requiring hospitalisation (with no hospitalisation during the 14 days prior to onset of pneumonia symptoms) and treatment with i.v. antibiotics for at least three days. Patients were also required to have at least two of the following: cough; purulent sputum production; rales or evidence of pulmonary consolidation; dyspnoea or tachypnoea; new onset hypoxaemia or requirement for mechanical ventilation. Patients were randomised in a 1:1 ratio to receive ceftobiprole, or ceftriaxone ± linezolid; all treatments were given i.v. Randomisation was stratified by Pneumonia Severity Index (PSI) score (<91 or ≥91) and need for anti-staphylococcal therapy at baseline. The primary endpoint was the clinical cure rate at the test-of-cure (TOC) visit, defined as either resolution of signs and symptoms of infection or sufficient improvement such that no further antibacterial therapy was necessary, and improvement or no adverse changes in findings on the chest radiograph.

The HAP study [ClinicalTrials.gov identifiers: NCT00210964, NCT00229008] was a multicentre, international, double-blind, non-inferiority study of adult patients with HAP undertaken at 157 centres between April 2005 and May 2007 [22]. Key inclusion criteria comprised: a clinical diagnosis of pneumonia after ≥72 h stay in hospital or a chronic care facility; clinical signs and symptoms of pneumonia; fever or leukocytosis/leukopenia; new or persistent radiographic infiltrates; and an Acute Physiology and Chronic Health Evaluation II (APACHE II) score between 8 and 25. Patients were randomised in a 1:1 ratio to receive ceftobiprole or ceftazidime plus linezolid, with all treatments given i.v. Randomisation was stratified by the presence of VAP (defined as pneumonia developing >48 h after onset of mechanical ventilation) and by APACHE II score (8–19 or 20–25). The primary endpoint was clinical cure at the TOC visit, defined as resolution of signs and symptoms of infection, or improvement to such an extent that no further antimicrobial therapy was necessary, in the absence of systemic non-study antibiotics.

Both studies were conducted in accordance with International Conference on Harmonization Guidelines for Good Clinical Practice, the Declaration of Helsinki, and applicable local regulations. Each study protocol was approved by an Independent Ethics Committee, and all patients provided written informed consent before any study procedures were carried out.

### Post hoc analysis

#### Patient population

The selection of risk factors was based on published literature demonstrating poorer outcomes in certain groups of pneumonia patients. For the CAP study, the high-risk group comprised patients with any of the following risk factors at baseline: Patient Outcome Research Team (PORT) risk score ≥III [23, 24]; aged ≥75 years [3]; sepsis [3]; chronic obstructive pulmonary disease (COPD) [3]; bacteraemia [3]; or treated in an intensive care unit (ICU) [25]. Patients from the HAP study who were included in the high-risk group comprised those with mechanical ventilation at any time during the study (but not VAP patients) [5, 26] or with any of the following at baseline: APACHE II score ≥15 [5, 27]; aged ≥75 years [28–30]; bacteraemia [5, 31]; treated in an ICU [32]; COPD [33]; and >10 comorbidities documented in the patient’s medical history [5, 34].

#### Study endpoints

Efficacy assessment of antimicrobial therapy has been traditionally based on the clinical response rate at a TOC visit after the treatment course (the primary endpoint in both of the CAP and HAP studies included here). However, in recent years, additional response assessments have emerged that may provide further clinically relevant insights into the efficacy of antimicrobial therapy. Based on evidence gathered from historical and modern studies of antibiotic therapy in CAP, the Foundation for the National Institutes of Health (FNIH) observed that antimicrobial treatment achieved symptom improvement by Day 3 after the start of treatment in approximately three-quarters of cases [35]. Accordingly, the FNIH recommended that symptom improvement at approximately three days after the start of treatment could be used as a relevant treatment response measure [35]. Recent regulatory guidance from the US Food and Drug Administration (FDA) endorsed early symptom improvement (as measured on Day 3–5) as a primary outcome measure in clinical trials investigating CAP [36]. The FNIH Biomarkers Consortium has not yet defined new endpoints for antimicrobial efficacy trials in HAP, but noted recently that a clinical response endpoint based on symptoms up to study Day 7 may be relevant [37].

Based on these recommendations, rate of early clinical response was used as the primary efficacy outcome measure in our analysis. Data collected at the first clinical assessment following baseline were used to assess early improvement (Day 3 in CAP and Day 4 in HAP). Additional outcome measures in high-risk patient groups were clinical cure at TOC and all-cause mortality, as well as safety and tolerability.

#### Study 1: Community-acquired pneumonia

Early clinical improvement was defined as clinical response at Day 3 after randomisation, as proposed by the FNIH [35]. Clinical response was defined as improvement or resolution of two or more symptoms (cough, pleuritic chest pain, dyspnoea and sputum production) and no worsening of other symptoms. The duration of i.v. therapy and the proportion of patients who, after three days, met the protocol-defined criteria for switch to oral cefuroxime were calculated post hoc.

The intention-to-treat (ITT) population included all randomised patients, excluding 28 randomised patients enrolled at a single study site (14 in each treatment arm), who were removed from the analysis due to significant deviations from the study protocol. The clinically evaluable (CE) population included all treated patients with a diagnosis of CAP, unless the duration of study drug therapy was less than 48 h or less than 80% of the intended dose, cure took place within <5 days, or if other pre-specified exclusion criteria applied.

Analyses were conducted in the overall high-risk group, as well in subgroups of patients with each of the following individual risk factors: PORT risk score ≥III; PORT risk score ≥IV; age ≥75 years; sepsis; COPD; bacteraemia; and treated in an ICU. Analyses were only performed when the numbers of patients in both treatment arms were 20 or above.

#### Study 2: Hospital-acquired pneumonia

Early clinical improvement was defined as clinical improvement at Day 4 after randomisation, based on grading ‘improved from baseline’, ‘unchanged from baseline’ or ‘worsened from baseline’ by the investigator. Patients were analysed according to whether or not they achieved a clinical response, defined as either clinical cure (as per the primary endpoint) or improvement of at least two symptoms according to an investigator assessment at Day 4.

The ITT population included all randomised patients. The CE population included patients who received at least one dose of study medication and were clinically evaluable at the TOC visit, excluding patients who received systemic non-study antibiotics for indications other than pneumonia.

Endpoints were assessed in the subgroup of patients with any high-risk factor, including mechanical ventilation at any time during the study (or ≤48 h prior to development of pneumonia) and any of the following occurring at baseline: APACHE II score ≥15; aged ≥75 years; bacteraemia; treated in an ICU; COPD; and >10 comorbidities. Further analyses were conducted in additional subgroups of patients defined by presence of each of these risk factors individually, but only when the number of patients in both treatment arms was 20 or above.

All post hoc analyses performed were exploratory. Therefore, endpoints were analysed descriptively with two-sided 95% confidence interval (CI) values for treatment difference, using a normal approximation, rather than with any formal statistical testing, and no *p* values were generated. Given the exploratory nature of the analyses and the lack of any formal statistical testing, no correction for multiple comparisons was applied. The post hoc analysis was performed using SAS version 9.3.

## Results

Results of the post hoc analysis are presented here for the CE population; corresponding data relating to the ITT population are provided in the Additional files 1, 2, 3, 4, 5, 6 and 7.

### Patient characteristics

From the CAP study, 469 patients were included in the CE population (all-patients group). Of these, 231 had been treated with ceftobiprole and 238 with ceftriaxone ± linezolid. The high-risk group included 193 patients treated with ceftobiprole and 205 patients treated with ceftriaxone ± linezolid. From the HAP study, 383 patients were included in the CE population (all-patients group); 198 patients were treated with ceftobiprole and 185 with ceftazidime plus linezolid. In total, 169 patients treated with ceftobiprole and 138 treated with ceftazidime plus linezolid were included in the high-risk group. The number of patients with each high-risk factor is provided in Table 1. As the number of CAP and HAP patients with bacteraemia at baseline was <20 in both treatment arms, further analyses were not carried out for this subgroup.

**Table 1:** Patients in high-risk subgroup categories (CE population)

CAP			HAP (excluding VAP)		
Baseline risk factor	Ceftobiprole	Ceftriaxone ± linezolid	Baseline risk factor	Ceftobiprole	Ceftazidime plus linezolid
Any risk factor	193	205	Any risk factor	169	138
PORT ≥ III	126	117	APACHE score ≥ 15	67	59
PORT ≥ IV	51	58	>10 comorbidities	63	61
Sepsis	123	135	Mechanical ventilation^a^	38	37
Bacteraemia^b^	7	14	Bacteraemia^b^	15	11
Age ≥ 75 years	39	50	Age ≥ 75 years	59	54
COPD	51	59	COPD	55	39
ICU	25	26	ICU	73	59

^a^Mechanical ventilation at baseline or at any point during the study

^b^Further analyses were not conducted in the bacteraemia group as the number of patients in both treatment arms was below 20

*APACHE* Acute Physiology and Chronic Health Evaluation, *CAP* community-acquired pneumonia, *CE* clinically evaluable, *COPD* chronic obstructive pulmonary disease, *HAP* hospital-acquired pneumonia, *ICU* intensive care unit, *PORT* Patient Outcome Research Team, *VAP* ventilator-associated pneumonia

The baseline characteristics for the all-patients groups (including both low- and high-risk patients) from both studies are provided in Additional file 1 and were previously described separately in detail [21, 22]. The baseline characteristics for the high-risk groups are provided in Table 2. In the high-risk CAP group, a higher proportion of patients had sepsis at baseline compared with the all-patients group (63.7–65.9% vs 53.2–56.7%). As expected, the percentage of patients aged ≥65 years was higher in the high-risk groups compared with the all-patients groups (CAP 44.9–45.6% vs 27.7–30.7%; HAP 62.3–62.7% vs 52.4–56.1%).

**Table 2:** Baseline characteristics for high-risk patients with CAP and HAP (excluding VAP) (CE population)

	High-risk CAP	
	Ceftobiprole (*n* = 193) n (%)	Ceftriaxone ± linezolid (*n* = 205) n (%)
Male	115 (59.6)	123 (60.0)
Age ≥ 65 years	88 (45.6)	92 (44.9)
Sepsis	123 (63.7)	135 (65.9)
Pre-study antibiotics within 24 h	97 (50.3)	121 (59.0)
Valid pathogen at baseline	59 (30.6)	68 (33.2)
Patients with linezolid use^a^	19 (9.8)	30 (14.6)
	High-risk HAP (excluding VAP)	
	Ceftobiprole (*n* = 169) n (%)	Ceftazidime plus linezolid (*n* = 138) n (%)
Male	117 (69.2)	80 (58.0)
Age ≥ 65 years	106 (62.7)	86 (62.3)
Sepsis	122 (72.2)	109 (79.0)
APACHE score ≥ 15	67 (39.6)	59 (42.8)
Ventilation at baseline	22 (13.0)	24 (17.4)
Pre-study antibiotics within 24 h	101 (59.8)	81 (58.7)
Valid pathogen at baseline	100 (59.2)	89 (64.5)
Anti-pseudomonal antibiotics^b^	24 (14.2)	16 (11.6)

^a^CAP patients suspected of MRSA infection received add-on linezolid if randomised to ceftriaxone; if randomised to ceftobiprole, they received add-on placebo instead of linezolid

^b^Empirical treatment with antibiotic therapy was added to the study treatment for 48 h in patients with a suspected infection due to *Pseudomonas aeruginosa* or for 5–7 days in patients with proven infection due to *Pseudomonas aeruginosa*

*APACHE* Acute Physiology and Chronic Health Evaluation, *CAP* community-acquired pneumonia, *CE* clinically evaluable, *HAP* hospital-acquired pneumonia, *MRSA* methicillin-resistant *Staphylococcus aureus, VAP* ventilator-associated pneumonia

In both the CAP and HAP high-risk groups, baseline characteristics were generally similar between patients in the ceftobiprole vs comparator arms, with a few notable differences (Table 2). Firstly, in the CAP high-risk group, the proportion of patients receiving add-on therapy for suspected MRSA was higher in the ceftriaxone ± linezolid arm (linezolid 14.6%) compared with the ceftobiprole arm (placebo 9.8%). Secondly, in the HAP high-risk group, there was a higher proportion of male patients in the ceftobiprole arm compared with the ceftazidime plus linezolid arm (69.2% vs 58.0%). Similarly, baseline characteristics were broadly similar for high-risk patients whether they had CAP or HAP (Table 2). However, a higher proportion of patients with HAP were aged ≥65 years (62.3–62.7% patients), compared with CAP patients (44.9–45.6% of patients). Additionally, the proportion of HAP patients with a valid pathogen at baseline was approximately double that observed in CAP patients (59.2–64.5% vs 30.6–33.2%).

In the overall high-risk CAP group, the majority of patients had a clinical improvement assessment at Day 3. Two patients (1.0%) in the ceftobiprole arm and three patients (1.5%) in the ceftriaxone ± linezolid arm did not have a Day 3 assessment. Of these five patients, three discontinued the study for reasons including withdrawal of informed consent (n = 1), study medication deemed ineffective (n = 1), and protocol deviation (n = 1). In the overall HAP high-risk group, all patients in the ceftobiprole arm had a Day 4 assessment. Seven patients (5.1%) in the ceftazidime plus linezolid arm did not have a Day 4 assessment, of whom six discontinued the study for reasons including adverse event (AE; n = 1), death (n = 3), clinical failure (n = 1) and discharge to a nursing home (n = 1). Patient characteristics for the ITT population are provided in Additional files 2–4.

### Clinical outcomes

#### Early clinical improvement

**Fig. 1:**
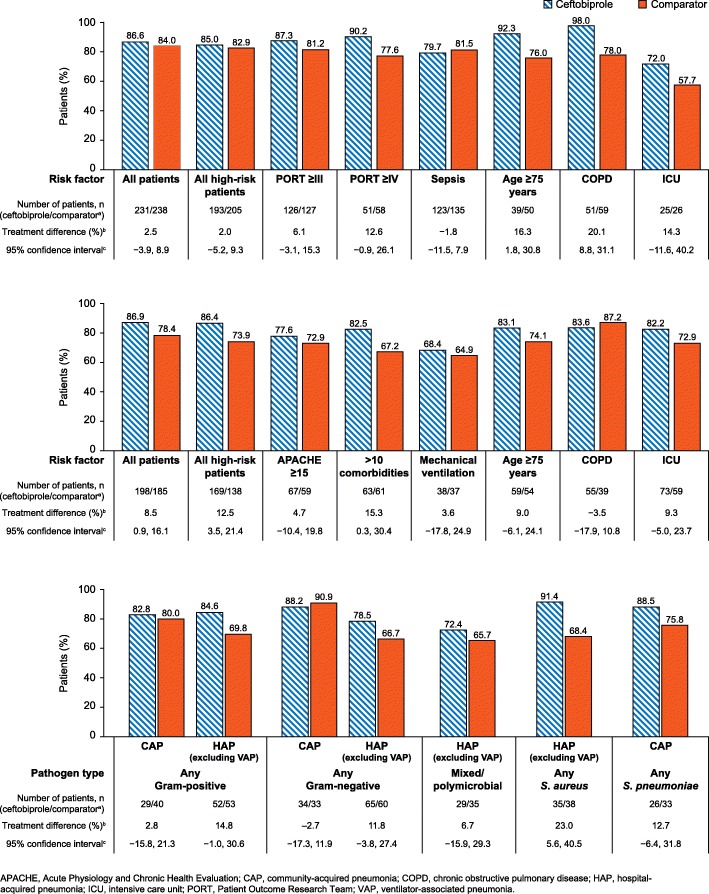
Early improvement in CAP or HAP patients, by risk factors and causative pathogen. **Top panel**. Early improvement at Day 3 in patients with CAP, by risk factor (CE population). **Middle panel**. Early improvement at Day 4 in patients with HAP (excluding VAP) by risk factor (CE population). **Lower panel**. Early improvement in high-risk group patients by pathogen type (CE population). ^a^The comparator treatment was ceftriaxone ± linezolid in CAP patients and ceftazidime plus linezolid in HAP (excluding VAP) patients. ^b^Between treatment difference calculated as ceftobiprole minus comparator ^c^Two-sided 95% confidence interval is based on a normal approximation to the difference of the two proportions. Analyses were not conducted in bacteraemia high-risk groups as the number of CAP and HAP patients in both treatment arms was <20. Early clinical improvement is defined as improved or cured at Day 3 in patients with CAP, and improved or cured at Day 4 in patients with HAP (excluding VAP). Early clinical improvements were evaluated by the investigator, based on an assessment of symptoms using standardised criteria

In patients with CAP the between-treatment difference in the proportion of patients with an early clinical improvement at Day 3 was <10%, in both the all-patients and the overall high-risk patient groups (Fig. 1a). When stratified by risk factor, between-treatment differences of >10% were observed in high-risk CAP patients aged 75 years or older, in patients with COPD at baseline, in ICU patients, and in patients with PORT risk score ≥4 (Fig. 1a). Each of these differences favoured ceftobiprole over ceftriaxone ± linezolid. Furthermore, in the subgroup of patients aged 75 years or older and in the subgroup of patients with COPD at baseline, these treatment differences were associated with 95% CI that did not cross zero (patients aged 75 years or older: treatment difference 16.3, 95% CI 1.8, 30.8; patients with COPD at baseline: treatment difference 20.1, 95% CI 8.8, 31.1).

In patients with HAP, a treatment difference of >10% in the percentage of patients with an early clinical improvement at Day 4 was observed in the overall high-risk patient group (Fig. 1b). This treatment difference (12.5%) was associated with 95% CI that did not cross zero (95% CI 3.5, 21.4). When stratified by risk factor (Fig. 1b), a between-treatment difference of >10% was observed in the subgroup of patients with >10 comorbidities at baseline. Again, this treatment difference (15.3%) favoured ceftobiprole over the comparator (ceftazidime plus linezolid) and the 95% CI did not cross zero (95% CI: 0.3, 30.4).

When stratified by causative pathogen, in the CAP study, a between-treatment difference of >10% in the proportion of high-risk patients with an early clinical improvement at Day 3 was observed in patients with any *S. pneumoniae* (Fig. 1c) (12.7%, favouring ceftobiprole; 95% CI − 6.4, 31.8). In the HAP study, between-treatment differences of >10% in the proportion of high-risk patients with early clinical improvement were observed in patients with any Gram-positive pathogen (14.8%), any Gram-negative pathogen (11.8%) or any *S. aureus* (23.0%). All of these treatment differences favoured ceftobiprole over ceftazidime plus linezolid. Furthermore, for the *S. aureus* group, the 95% CIs did not include zero (5.6, 40.5).

In the ITT population, no between-treatment differences of >10% were observed in the proportion of CAP or HAP patients with an early clinical improvement in the all-patients and high-risk groups. When stratified by risk factor and causative pathogen, in the CAP study, treatment differences of >10% in early response were observed in the PORT ≥IV group (11.9% favouring ceftobiprole; 95% CI − 1.2, 25.0) and in patients with any Gram-negative pathogen (− 11.4% favouring ceftriaxone ± linezolid; 95% CI − 26.0, 3.3) (Additional file 6). In the HAP study, treatment differences of >10% were observed in patients with >10 comorbidities (11.9%, favouring ceftobiprole; 95% CI − 1.4, 24.9), in patients with any MRSA (14.9%, favouring ceftobiprole; 95% CI − 9.1, 38.8) and in patients with any *P. aeruginosa* (14.8%, favouring ceftobiprole; 95% CI − 9.2, 38.9).

#### Clinical cure at TOC visit

There were no treatment differences of >10% in the proportion of CAP and HAP patients achieving a clinical cure at the TOC visit, for both the all-patients and the high-risk groups (Table 3). When analysed by causative pathogen and risk factor, treatment differences of >10% in clinical cure at TOC were observed in ICU patients in the CAP study (10.5%, favouring ceftobiprole; 95% CI − 15.2, 36.1) and patients receiving mechanical ventilation in the HAP study (14.7%, favouring ceftobiprole; 95% CI − 7.6, 37.1) (Table 3).

**Table 3:** Clinical cure at TOC visit by high-risk factor and pathogen type (CE population)

	Number of patients (ceftobiprole/ comparator)	Clinical cure at TOC (%, ceftobiprole/ comparator)	Treatment difference (%)^a^	95% CI^b^
All patients (CAP)	231/238	86.6/87.4	−0.8	−6.9, 5.3
High-risk patients (CAP)	193/205	86.0/86.8	−0.8	−7.6, 5.9
Any Gram-positive	29/40	89.7/90.0	−0.3	−14.8, 14.1
Any Gram-negative	34/33	82.4/90.9	−8.6	−24.7, 7.6
Any *S. pneumoniae*	26/33	92.3/90.9	1.4	−12.8, 15.6
PORT ≥ III	126/117	86.5/86.3	0.2	−8.4, 8.8
PORT ≥ IV	51/58	90.2/84.5	5.7	−6.7, 18.1
Sepsis	123/135	84.6/86.7	−2.1	−10.7, 6.5
Age ≥ 75 years	39/50	92.3/86.0	6.3	−6.4, 19.1
COPD	51/59	86.3/86.4	−0.2	−13.0, 12.7
ICU	25/26	72.0/61.5	10.5	−15.2, 36.1
All patients (HAP, excl. VAP)	198/185	77.8/76.2	1.6	−6.9, 10.0
High-risk patients (HAP, excl. VAP)	169/138	75.7/71.7	4.0	−5.9, 13.9
Any Gram-positive	52/53	69.2/69.8	−2.5	−19.9, 15.0
Any Gram-negative	65/60	67.7/73.3	−5.6	−21.6, 10.3
Mixed/polymicrobial	29/35	62.1/68.6	−6.5	−29.9, 16.9
Any *S. aureus*	35/38	68.6/71.1	−2.5	− 23.6, 18.6
APACHE score ≥ 15	67/59	68.7/64.4	4.2	−12.3, 20.8
>10 comorbidities	63/61	73.0/67.2	5.8	−10.3, 21.9
Mechanical ventilation	38/37	55.3/40.5	14.7	−7.6, 37.1
Age ≥ 75 years	59/54	72.9/77.8	−4.9	−20.8, 11.0
COPD	55/39	83.6/76.9	6.7	−9.7, 23.2
ICU	73/59	69.9/66.1	3.8	−12.3, 19.8

^a^Between treatment difference calculated as ceftobiprole minus ceftriaxone ± linezolid for patients with CAP, and ceftobiprole minus ceftazidime plus linezolid for patients with HAP (excluding VAP)

^b^Two-sided 95% CI is based on a normal approximation to the difference of the two proportions

*APACHE* Acute Physiology and Chronic Health Evaluation, *CAP* community-acquired pneumonia, *CE* clinically evaluable, *CI* confidence interval, *COPD* chronic obstructive pulmonary disease, *HAP* hospital-acquired pneumonia, *ICU* intensive care unit, *MRSA* methicillin-resistant *Staphylococcus aureus, PORT* Patient Outcome Research Team, *TOC* test-of-cure, *VAP*, ventilator-associated pneumonia

Similarly, in the ITT population, treatment differences in the proportion of CAP and HAP patients achieving a clinical cure at the TOC visit were mostly ≤10% (Additional file 5). Exceptions, both in the HAP study, included patients with mixed or polymicrobial infections (treatment difference − 11.8%, favouring ceftazidime plus linezolid; 95% CI − 32.0, 8.3) and patients with COPD (treatment difference 10.6%, favouring ceftobiprole; 95% CI − 5.1, 26.4).

#### 30-day all-cause mortality

**Fig. 2:**
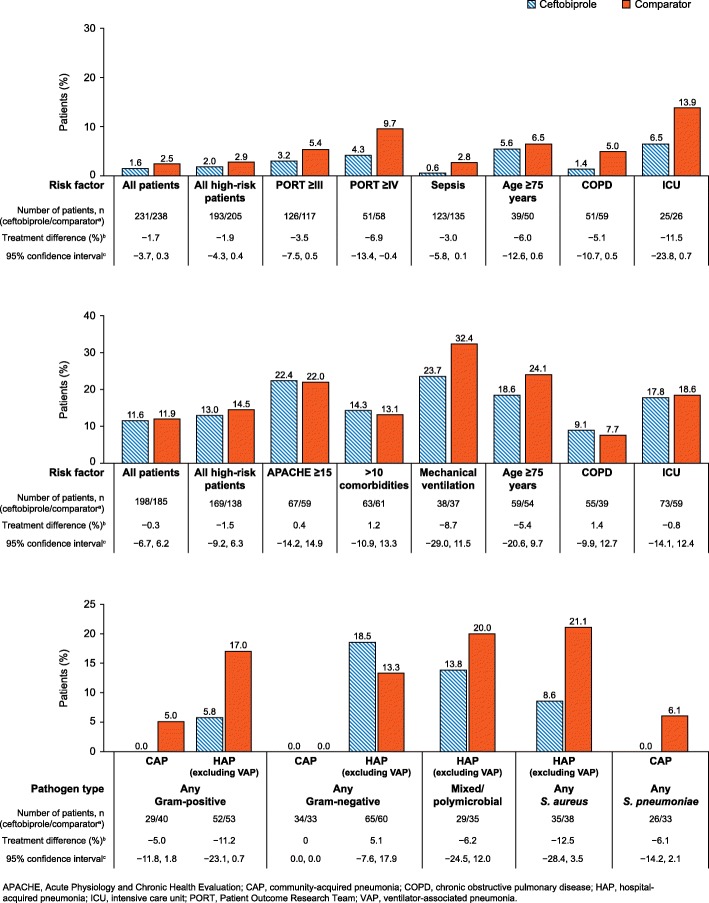
30-day all-cause mortality in CAP or HAP patients, by risk factors and causative pathogen. **Top panel**. 30-day all-cause mortality in patients with CAP, by risk factor (CE population). **Middle panel**. 30-day all-cause mortality in patients with HAP (excluding VAP) by risk factor (CE population). **Lower panel**. 30-day all-cause mortality in high-risk group patients by pathogen type (CE population). ^a^The comparator treatment was ceftriaxone ± linezolid in CAP patients and ceftazidime plus linezolid in HAP (excluding VAP) patients. ^b^Between treatment difference calculated as ceftobiprole minus comparator. ^c^Two-sided 95% confidence interval is based on a normal approximation to the difference of the two proportions

Overall, no between-treatment differences of >10% were observed in 30-day all-cause mortality in CAP and HAP patients, for both the all-patients and high-risk groups (Fig. 2a; Fig. 2b). When stratified by risk factor, a between-treatment difference of >10% in 30-day all-cause mortality was observed in CAP patients treated in the ICU (− 11.5%; favouring ceftriaxone ± linezolid; 95% CI − 23.8, 0.7) (Fig. 2a). No between-treatment differences in all-cause mortality of >10% were observed in high-risk HAP patients when analysed by risk factor (Fig. 2b).

When analysed by causative pathogen, between-treatment differences in mortality rate of >10% were observed in high-risk HAP patients with any Gram-positive pathogen (− 11.2%; favouring ceftazidime plus linezolid; 95% CI − 23.1, 0.7) or with any *S. aureus* (− 12.5%; favouring ceftazidime plus linezolid; 95% CI − 28.4, 3.5) (Fig. 2c).

In the ITT population, there were no between-treatment differences of >10% in 30-day all-cause mortality for both CAP and HAP patients, in the all-patients and high-risk groups. When stratified by risk factor, a between-treatment difference of >10% in 30-day all-cause mortality was observed in patients with bacteraemia in the HAP study (− 16.2%, favouring ceftazidime plus linezolid; 95% CI − 40.6, 8.2). When stratified by causative pathogen, a between-treatment difference of >10% in 30-day all-cause mortality was observed in patients with any Gram-positive pathogen in the HAP study (− 11.5%, favouring ceftazidime plus linezolid; 95% CI − 24.2, 1.3) (Additional file 7).

#### Safety

Safety findings in the all-patients groups from both studies have previously been reported and discussed in detail [21, 22]. In the high-risk populations included here, the incidence of treatment-emergent AEs was broadly similar in CAP and HAP patients (Table 4). A higher proportion of patients with HAP reported serious AEs (SAEs) compared with CAP patients, in both the all-patients and high-risk groups (Table 4).

**Table 4:** Summary of treatment-emergent AEs (CE population)

Number of patients with ≥ 1, n (%)	All patients				High-risk			
	CAP		HAP (excluding VAP)		CAP		HAP (excluding VAP)	
	Ceftobiprole (*n* = 231)	Ceftriaxone ± linezolid (*n* = 238)	Ceftobiprole (*n* = 198)	Ceftazidime plus linezolid (*n* = 185)	Ceftobiprole (*n* = 193)	Ceftriaxone ± linezolid (*n* = 205)	Ceftobiprole (*n* = 169)	Ceftazidime plus linezolid (*n* = 138)
AE	163 (70.6)	149 (62.6)	141 (71.2)	140 (75.7)	138 (71.5)	133 (64.9)	126 (74.6)	112 (81.2)
SAE	23 (10.0)	24 (10.1)	53 (26.8)	39 (21.1)	21 (10.9)	22 (10.7)	51 (30.2)	33 (23.9)
Treatment-related AE	82 (35.5)	61 (25.6)	51 (25.8)	49 (26.5)	70 (36.3)	57 (27.8)	46 (27.2)	43 (31.2)
Treatment-related SAE	2 (0.9)	3 (1.3)	7 (3.5)	3 (1.6)	2 (1.0)	2 (1.0)	7 (4.1)	3 (2.2)
SAE leading to death	3 (1.3)	6 (2.5)	27 (13.6)	28 (15.1)	3 (1.6)	6 (2.9)	26 (15.4)	25 (18.1)

*AE* adverse event, *CAP* community-acquired pneumonia, *CE* clinically evaluable, *HAP* hospital-acquired pneumonia, *SAE* serious adverse event, *VAP* ventilator-associated pneumonia

The proportions of ceftobiprole-treated CAP patients experiencing AEs, SAEs, treatment-related AEs, treatment-related SAEs and AEs leading to death were similar in the all-patients and high-risk groups (Table 4). In the ceftobiprole arm, AEs were reported by 71.5% of high-risk patients vs 70.6% of all patients, with SAEs reported by 10.9 and 10.0% of all patients and high-risk patients, respectively. Treatment-related AEs and SAEs were reported by 36.3% of high-risk patients vs 35.5% of all patients and 1.0% of high-risk patients vs 0.9% of all patients, respectively. AEs leading to death occurred in 1.6% of high-risk patients vs 1.3% of all patients.

In the ceftobiprole arm, the proportion of high-risk HAP patients reporting AEs, SAEs, treatment-related AEs, treatment-related SAEs and AEs leading to death was comparable with the all-patients group (Table 4). AEs were reported by 74.6% of high-risk patients vs 71.2% of all patients. A comparable proportion of HAP patients in the high-risk group and all-patients group reported SAEs (30.2% vs 26.8%), related AEs (27.8% vs 25.8%), and SAEs leading to death (15.4% vs 13.6%).

The safety profiles of ceftobiprole and the comparator treatments were broadly similar, with some minor differences (Table 4). In high-risk CAP patients, a higher proportion of patients receiving ceftobiprole reported treatment-emergent AEs, compared with patients receiving ceftriaxone ± linezolid (71.5% vs 64.9%). However, in high-risk HAP patients, a higher proportion of patients receiving ceftazidime plus linezolid reported treatment-emergent AEs compared with patients receiving ceftobiprole (81.2% vs 74.6%). In high-risk HAP patients, the incidence of treatment-emergent SAEs was higher in patients receiving ceftobiprole compared with patients receiving ceftazidime plus linezolid (30.2% vs 23.9%). In high-risk CAP patients, the incidence of treatment-emergent SAEs was comparable between the treatment groups (10.9% vs 10.7%).

## Discussion

The results of this exploratory post hoc analysis of two large randomised controlled trials indicate that ceftobiprole treatment is effective in severely ill patients with pneumonia at risk of poor outcomes. In high-risk patients with CAP or HAP, ceftobiprole treatment demonstrated similar results to the comparator treatment (ceftriaxone ± linezolid in CAP and ceftazidime plus linezolid in HAP patients) in terms of early clinical improvement, clinical cure at TOC, and all-cause mortality. Furthermore, in high-risk patients with HAP, a higher percentage of patients had early clinical improvement in the ceftobiprole group compared with ceftazidime plus linezolid treatment (between-treatment difference: 12.5% [95% CI: 3.5, 21.4]).

Potential for improved clinical outcomes with ceftobiprole compared with the active-control therapies was observed in several high-risk patient subgroups. A higher proportion of ceftobiprole-treated CAP patients aged ≥75 years or with COPD at baseline, and HAP patients with >10 baseline comorbidities had early clinical response compared with patients who received the active-control therapy. Overall, these findings suggest that the rapid bactericidal action of ceftobiprole [38] may have advantages over other cephalosporins in high-risk patients with HAP (excluding VAP) and in some subgroups of high-risk patients with CAP, in whom rapid improvement is urgently required to ensure better outcomes.

These results are timely, given that recent guidance documents produced by the FNIH and the FDA have recommended early symptom improvement (FNIH: 3 days after the start of treatment; FDA: 3–5 days) may be a useful measure of treatment response in CAP [35, 36]. Such measures may also be useful in HAP, as the FNIH Biomarkers Consortium has noted recently that definitions of response based on symptoms up to study Day 7 may be relevant [37]. However, a firm consensus on this point has not yet been reached.

These findings build on the results reported in the original publications. Notably, the early improvement observed in high-risk HAP patients was also observed in the full population; in the CE population, a higher proportion of HAP (excluding VAP) patients in the ceftobiprole group showed early improvement at Day 4 compared with the ceftazidime/linezolid group (86.9% vs 78.4%; treatment difference 8.5 [95% CI: 0.9, 16.1]) [22]. In both trials, ceftobiprole was generally as effective as the comparator across risk groups in terms of clinical cure at TOC [21, 22].

Few differences in clinical outcomes were observed between treatments when analysed by causative pathogen type, with the exception of high-risk HAP patients with any *S. aureus* pathogen. In this subgroup, a higher proportion of patients treated with ceftobiprole had early improvement at Day 4 compared with the comparator treatment.

The results of this post hoc analysis confirm the initial safety results from these Phase III trials, which demonstrated that ceftobiprole treatment for CAP and HAP is well-tolerated, with a safety profile that is consistent with other cephalosporins [21, 22]. The incidence of AEs in the CAP and HAP high-risk group was similar to that observed in the all-patients group, suggesting that the safety profile of ceftobiprole treatment is not altered in high-risk CAP and HAP patients.

Baseline characteristics were similar in high-risk CAP and HAP patients included in this post hoc analysis. This similarity demonstrates that no significant differences exist between high-risk HAP and CAP patients in terms of underlying characteristics and risk factors, and that the population studied can be considered as fairly homogenous.

Several limitations of this exploratory post hoc analysis need to be taken into consideration when interpreting the results. Notably, the sample size was relatively small, especially in some of the subgroup analyses of individual risk factors. In addition, no formal hypothesis testing was planned or undertaken, and no correction was made for the multiple comparisons performed, which, together with the small sample size, increased the risk of chance findings. Furthermore, the original studies were not powered to detect statistical treatment differences between subgroups of patients. The results of the post hoc analysis therefore need to be interpreted with caution. Another limitation to be considered is that the original studies of ceftobiprole in CAP and HAP patients included a highly controlled patient population, in order to allow a comparison of ceftobiprole with the reference treatment. Although the population included in the HAP study was noted to be representative of nosocomial pneumonia patients in terms of age, underlying conditions and severity of disease, the patients included in this post hoc analysis may not be fully representative of a ‘real-life’ population [22].

## Conclusions

Ceftobiprole appears to be an efficacious and generally well-tolerated therapy for patients with pneumonia who are severely ill or at high risk of poor outcomes. The results of this study, which analysed the clinically evaluable population, provide preliminary evidence that ceftobiprole may be associated with early improvement in these patient groups. Particularly notable results seeming to favour ceftobiprole over comparators were observed in high-risk patients with HAP (excluding VAP) and in some subgroups of high-risk patients with CAP, such as those aged ≥75 years or with COPD. Given the exploratory nature of these analyses, the results should be interpreted with caution.

## Additional files

Additional file 1: **Table S1.** Baseline characteristics for the all-patients groups for CAP and HAP (CE population). (DOCX 22 kb)

Additional file 2: **Table S2.** Patients in high-risk subgroup categories (ITT population). (DOCX 13 kb)

Additional file 3: **Table S3.** Baseline characteristics for high-risk patients with CAP and HAP (excluding VAP) (ITT population). (DOCX 13 kb)

Additional file 4: **Table S4.** Baseline characteristics for CAP and HAP all-patients group (ITT population). (DOCX 13 kb)

Additional file 5: **Table S5.** Clinical cure at TOC visit by high-risk factor and pathogen type (ITT population) (DOCX 14 kb)

Additional file 6: **Figure S1.** Early improvement (ITT population). a. Early improvement at Day 3 in patients with CAP, by risk factor (ITT population). b. Early improvement at Day 4 in patients with HAP (excluding VAP) by risk factor (ITT population). c. Early improvement in high-risk group patients by pathogen type (ITT population). ^a^The comparator treatment was ceftriaxone ± linezolid in CAP patients and ceftazidime plus linezolid in HAP (excluding VAP) patients. ^b^Between treatment difference calculated as ceftobiprole minus comparator. ^c^Two-sided 95% confidence interval is based on a normal approximation to the difference of the two proportions. Early clinical improvement is defined as improved or cured at Day 3 in patients with CAP, and improved or cured at Day 4 in patients with HAP (excluding VAP). Early clinical improvements were evaluated by the investigator, based on an assessment of symptoms using standardised criteria. (PDF 681 kb)

Additional file 7: **Figure S2.** 30-day all-cause mortality (ITT population). a. 30-day all-cause mortality in patients with CAP, by risk factor (ITT population). b. 30-day all-cause mortality in patients with HAP (excluding VAP) by risk factor (ITT population). c. 30-day all-cause mortality in high-risk group patients by pathogen type (ITT population). ^a^The comparator treatment was ceftriaxone ± linezolid in CAP patients and ceftazidime plus linezolid in HAP (excluding VAP) patients. ^b^Between treatment difference calculated as ceftobiprole minus comparator. ^c^Two-sided 95% confidence interval is based on a normal approximation to the difference of the two proportions. (PDF 667 kb)

## Abbreviations

AE: Adverse event
APACHE: Acute Physiology and Chronic Health Evaluation
CAP: Community-acquired pneumonia
CE: Clinically evaluable
CI: Confidence interval
COPD: Chronic obstructive pulmonary disease
FDA: Food and Drug Administration
FNIH: Foundation for the National Institutes of Health
HAP: Hospital-acquired pneumonia
i.v.: Intravenous
ICU: Intensive care unit
ITT: Intention-to-treat
MRSA: Methicillin-resistant *Staphylococcus aureus*
PORT: Patient Outcome Research Team
PSI: Pneumonia severity index
SAE: Serious adverse event
TOC: Test-of-cure
VAP: Ventilator-associated pneumonia
