# Supplementary material for: Early improvement in severely ill patients with pneumonia treated with ceftobiprole: a retrospective analysis of two major trials
Source: BMC Infect Dis. 2019 Feb 26;19:195. doi: 10.1186/s12879-019-3820-y (PMC6390565; doi:10.1186/s12879-019-3820-y)
Supplement: Supplementary file 1 — Table S1. Baseline characteristics for the all-patients groups for CAP and HAP (CE population). (DOCX 22 kb) [file 12879_2019_3820_MOESM1_ESM.docx]

**Additional file 1**

Baseline characteristics for the all-patients groups for CAP and HAP (CE population)

|  | **CAP patients** | |
| --- | --- | --- |
|  | **Ceftobiprole (n=231)**  **n (%)** | **Ceftriaxone ± linezolid (n=238)**  **n (%)** |
| Male | 135 (58.4) | 139 (58.4) |
| Age ≥65 years | 90 (39.0) | 97 (40.8) |
| Sepsis | 123 (53.2) | 135 (56.7) |
| Pre-study antibiotics within 24 hours | 120 (51.9) | 137 (57.6) |
| Valid pathogen at baseline | 68 (29.4) | 76 (31.9) |
| Patients with linezolid use^a^ | 23 (10.0) | 34 (14.3) |
|  | **HAP (excluding VAP) patients** | |
|  | **Ceftobiprole**  **(n=198)**  **n (%)** | **Ceftazidime plus linezolid**  **(n=185)**  **n (%)** |
| Male | 139 (70.2) | 112 (60.5) |
| Age ≥65 years | 111 (56.1) | 97 (52.4) |
| Sepsis | 146 (73.7) | 147 (79.5) |
| APACHE score ≥15 | 67 (33.8) | 59 (31.9) |
| Ventilation at baseline | 22 (11.1) | 24 (13.0) |
| Pre-study antibiotics within 24 hours | 112 (56.6) | 103 (55.7) |
| Valid pathogen at baseline | 117 (59.1) | 120 (64.9) |
| Anti-pseudomonal antibiotics^b^ | 27 (13.6) | 19 (10.3) |

^a^CAP patients suspected of MRSA infection received add-on linezolid if randomised to ceftriaxone; if randomised to ceftobiprole, they received add-on placebo instead of linezolid.

^b^Empirical treatment with antibiotic therapy was added to the study treatment for 48 hours in patients with a suspected infection due to *Pseudomonas aeruginosa* or for 5–7 days in patients with proven infection due *to Pseudomonas aeruginosa*.
APACHE, Acute Physiology and Chronic Health Evaluation; CAP, community-acquired pneumonia; CE, clinically evaluable; HAP, hospital-acquired pneumonia; MRSA, methicillin-resistant *Staphylococcus aureus*; VAP, ventilator-associated pneumonia.
