# Supplementary material for: Early improvement in severely ill patients with pneumonia treated with ceftobiprole: a retrospective analysis of two major trials
Source: BMC Infect Dis. 2019 Feb 26;19:195. doi: 10.1186/s12879-019-3820-y (PMC6390565; doi:10.1186/s12879-019-3820-y)
Supplement: Supplementary file 2 — Table S2. Patients in high-risk subgroup categories (ITT population). (DOCX 13 kb) [file 12879_2019_3820_MOESM2_ESM.docx]

**Additional file 2**Patients in high-risk subgroup categories (ITT population)

| **CAP** | | | **HAP (excluding VAP)** | | |
| --- | --- | --- | --- | --- | --- |
| **Baseline risk factor** | **Ceftobiprole** | **Ceftriaxone ± linezolid** | **Baseline risk factor** | **Ceftobiprole** | **Ceftazidime plus linezolid** |
| **Any risk factor** | 253 | 276 | **Any risk factor** | 244 | 230 |
| **PORT ≥III** | 158 | 149 | **APACHE score ≥15** | 101 | 104 |
| **PORT ≥IV** | 69 | 72 | **>10 comorbidities** | 82 | 92 |
| **Sepsis** | 164 | 178 | **Mechanical ventilation^a^** | 69 | 70 |
| **Bacteraemia**^b^ | 11 | 17 | **Bacteraemia** | 24 | 27 |
| **Age ≥75 years** | 54 | 62 | **Age ≥75 years** | 78 | 88 |
| **COPD** | 70 | 80 | **COPD** | 76 | 66 |
| **ICU** | 31 | 36 | **ICU** | 111 | 108 |

^a^Mechanical ventilation at baseline or at any point during the study. ^b^Further analyses were not conducted in the bacteraemia group as the number of patients in both treatment arms was below 20.

APACHE, Acute Physiology and Chronic Health Evaluation; CAP, community-acquired pneumonia; COPD, chronic obstructive pulmonary disease; HAP, hospital-acquired pneumonia; ICU, intensive care unit; ITT, intention-to-treat; PORT, Patient Outcome Research Team; VAP, ventilator-associated pneumonia.
