# Supplementary material for: Early improvement in severely ill patients with pneumonia treated with ceftobiprole: a retrospective analysis of two major trials
Source: BMC Infect Dis. 2019 Feb 26;19:195. doi: 10.1186/s12879-019-3820-y (PMC6390565; doi:10.1186/s12879-019-3820-y)
Supplement: Supplementary file 3 — Table S3. Baseline characteristics for high-risk patients with CAP and HAP (excluding VAP) (ITT population). (DOCX 13 kb) [file 12879_2019_3820_MOESM3_ESM.docx]

**Additional file 3**Baseline characteristics for high-risk patients with CAP and HAP (excluding VAP) (ITT population)

|  | **High-risk CAP** | |
| --- | --- | --- |
|  | **Ceftobiprole (n=253)**  **n (%)** | **Ceftriaxone ± linezolid (n=276)**  **n (%)** |
| Male | 149 (58.9) | 162 (58.7) |
| Age ≥65 years | 108 (42.7) | 119 (43.1) |
| Sepsis | 164 (64.8) | 178 (64.5) |
| Pre-study antibiotics within 24 hours | 134 (53.0) | 163 (59.1) |
| Valid pathogen at baseline | 74 (29.2) | 87 (31.5) |
| Patients with linezolid use^a^ | 25 (9.9) | 36 (13.0) |
|  | **High-risk HAP (excluding VAP)** | |
|  | **Ceftobiprole**  **(n=244)**  **n (%)** | **Ceftazidime plus linezolid**  **(n=230)**  **n (%)** |
| Male | 172 (70.5) | 133 (57.8) |
| Age ≥65 years | 141 (57.8) | 138 (60.0) |
| Sepsis | 182 (74.6) | 181 (78.7) |
| APACHE score ≥15 | 101 (41.4) | 104 (45.2) |
| Ventilation at baseline | 41 (16.8) | 44 (19.1) |
| Pre-study antibiotics within 24 hours | 153 (62.7) | 149 (64.8) |
| Valid pathogen at baseline | 151 (61.9) | 145 (63.0) |
| Anti-pseudomonal antibiotics^b^ | 33 (13.5) | 28 (12.2) |

^a^CAP patients suspected of MRSA infection received add-on linezolid if randomised to ceftriaxone; if randomised to ceftobiprole, they received add-on placebo instead of linezolid.

^b^Empirical treatment with antibiotic therapy was added to the study treatment for 48 hours in patients with a suspected infection due to *Pseudomonas aeruginosa* or for 5–7 days in patients with proven infection due *to Pseudomonas aeruginosa*.
APACHE, Acute Physiology and Chronic Health Evaluation; CAP, community-acquired pneumonia; HAP, hospital-acquired pneumonia; ITT, intention-to-treat; MRSA, methicillin-resistant *Staphylococcus aureus*; VAP, ventilator-associated pneumonia.
