# Supplementary material for: Early improvement in severely ill patients with pneumonia treated with ceftobiprole: a retrospective analysis of two major trials
Source: BMC Infect Dis. 2019 Feb 26;19:195. doi: 10.1186/s12879-019-3820-y (PMC6390565; doi:10.1186/s12879-019-3820-y)
Supplement: Supplementary file 4 — Table S4. Baseline characteristics for CAP and HAP all-patients group (ITT population). (DOCX 13 kb) [file 12879_2019_3820_MOESM4_ESM.docx]

**Additional file 4**Baseline characteristics for CAP and HAP all-patients group (ITT population)

|  | **CAP patients** | |
| --- | --- | --- |
|  | **Ceftobiprole (n=314)**  **n (%)** | **Ceftriaxone ± linezolid (n=324)**  **n (%)** |
| Male | 179 (57.0) | 187 (57.7) |
| Age ≥65 years | 111 (35.4) | 126 (38.9) |
| Sepsis | 164 (52.2) | 178 (54.9) |
| Pre-study antibiotics within 24 hours | 168 (53.5) | 186 (57.4) |
| Valid pathogen at baseline | 87 (27.7) | 97 (29.9) |
| Patients with linezolid use^a^ | 30 (9.6) | 40 (12.3) |
|  | **HAP (excluding VAP) patients** | |
|  | **Ceftobiprole**  **(n=287)**  **n (%)** | **Ceftazidime plus linezolid**  **(n=284)**  **n (%)** |
| Male | 202 (70.4) | 170 (59.9) |
| Age ≥65 years | 152 (53.0) | 151 (53.2) |
| Sepsis | 217 (75.6) | 226 (79.6) |
| APACHE score ≥15 | 101 (35.2) | 104 (36.6) |
| Ventilation at baseline | 41 (14.3) | 44 (15.5) |
| Pre-study antibiotics within 24 hours | 169 (58.9) | 176 (62.0) |
| Valid pathogen at baseline | 179 (62.4) | 181 (63.7) |
| Anti-pseudomonal antibiotics^b^ | 37 (12.9) | 31 (10.9) |

^a^CAP patients suspected of MRSA infection received add-on linezolid if randomised to ceftriaxone; if randomised to ceftobiprole, they received add-on placebo instead of linezolid.

^b^Empirical treatment with antibiotic therapy was added to the study treatment for 48 hours in patients with a suspected infection due to *Pseudomonas aeruginosa* or for 5–7 days in patients with proven infection due *to Pseudomonas aeruginosa*.
APACHE, Acute Physiology and Chronic Health Evaluation; CAP, community-acquired pneumonia; HAP, hospital-acquired pneumonia; ITT, intention-to-treat; MRSA, methicillin-resistant *Staphylococcus aureus*; VAP, ventilator-associated pneumonia.
