# Supplementary material for: Early improvement in severely ill patients with pneumonia treated with ceftobiprole: a retrospective analysis of two major trials
Source: BMC Infect Dis. 2019 Feb 26;19:195. doi: 10.1186/s12879-019-3820-y (PMC6390565; doi:10.1186/s12879-019-3820-y)
Supplement: Supplementary file 5 — Table S5. Clinical cure at TOC visit by high-risk factor and pathogen type (ITT population) (DOCX 14 kb) [file 12879_2019_3820_MOESM5_ESM.docx]

**Additional file 5**Clinical cure at TOC visit by high-risk factor and pathogen type (ITT population)

|  | **Number of patients**  **(ceftobiprole/ comparator)** | **Clinical cure at TOC**  **(%, ceftobiprole/ comparator)** | **Treatment difference (%)^a^** | **95% CI^b^** |
| --- | --- | --- | --- | --- |
| **All patients (CAP)** | **314 / 324** | **76.4 / 79.3** | **−2.9** | **−9.3, 3.6** |
| **High-risk (CAP)** | **253 / 276** | **76.7 / 79.0** | **−2.3** | **−9.4, 4.8** |
| Any Gram-positive | 40 / 49 | 72.5 / 77.6 | −5.1 | −23.2, 13.1 |
| Any Gram-negative | 44 / 44 | 77.3 / 84.1 | −6.8 | −23.3, 9.6 |
| Any *S. pneumoniae* | 32 / 37 | 81.3 / 83.8 | −2.5 | −20.5, 15.5 |
| PORT ≥III | 158 / 149 | 79.1 / 78.5 | 0.6 | −8.6, 9.7 |
| PORT ≥IV | 69 / 72 | 81.2 / 77.8 | 3.4 | −9.9, 16.7 |
| Sepsis | 164 / 178 | 76.8 / 80.9 | −4.1 | −12.7, 4.6 |
| Age ≥75 years | 54 / 62 | 81.5 / 79.0 | 2.4 | −12.0, 16.9 |
| COPD | 70 / 80 | 71.4 / 73.8 | −2.3 | −16.6, 12.0 |
| ICU | 31 / 36 | 58.1 / 50.0 | 8.1 | −15.8, 31.9 |
| **All patients (HAP)** | **287 / 284** | **59.6 / 58.8** | **0.8** | **−7.3, 8.8** |
| **High-risk (HAP)** | **244 / 230** | **57.8 / 52.6** | **5.2** | **−3.8, 14.1** |
| Any Gram-positive | 73 / 86 | 53.4 / 52.3 | 1.1 | −14.5, 16.7 |
| Any Gram-negative | 105 / 91 | 50.5 / 60.4 | −10.0 | −23.8, 3.9 |
| Mixed/polymicrobial | 46 / 47 | 43.5 / 55.3 | −11.8 | −32.0, 8.3 |
| Any *S. aureus* | 49 / 65 | 51.0 / 52.3 | −1.3 | −19.8, 17.2 |
| Any MRSA pathogen | 26 / 29 | 50.0 / 44.8 | 5.2 | −21.2, 31.6 |
| Any *P. aeruginosa* pathogen | 27 / 27 | 51.9 / 51.9 | 0 | −26.7, 26.7 |
| APACHE score ≥15 | 101 / 104 | 49.5 / 46.2 | 3.4 | −10.3, 17.0 |
| >10 comorbidities | 82 / 92 | 61.0 / 52.2 | 8.8 | −5.9, 23.5 |
| Mechanical ventilation | 69 / 70 | 30.4 / 27.1 | 3.3 | −11.8, 18.3 |
| Age ≥75 years | 78 / 88 | 59.0 / 56.8 | 2.2 | −12.9, 17.2 |
| Bacteraemia at baseline | 24 / 27 | 29.2 / 29.6 | −0.5 | −25.5, 24.6 |
| COPD | 76 / 66 | 69.7 / 59.1 | 10.6 | −5.1, 26.4 |
| ICU | 111 / 108 | 52.3 / 45.4 | 6.9 | −6.3, 20.1 |

^a^Between treatment difference calculated as ceftobiprole minus ceftriaxone ± linezolid for patients with CAP, and ceftobiprole minus ceftazidime + linezolid for patients with HAP (excluding VAP). ^b^Two-sided 95% confidence interval is based on a normal approximation to the difference of the two proportions.
APACHE, Acute Physiology and Chronic Health Evaluation; CAP, community-acquired pneumonia; CI, confidence interval; COPD, chronic obstructive pulmonary disease; HAP, hospital-acquired pneumonia; ICU, intensive care unit; ITT, intention-to-treat; MRSA, methicillin-resistant *Staphylococcus aureus*; PORT, Patient Outcome Research Team; TOC, test-of-cure; VAP, ventilator-associated pneumonia.
