# Supplementary material for: Early improvement in severely ill patients with pneumonia treated with ceftobiprole: a retrospective analysis of two major trials
Source: BMC Infect Dis. 2019 Feb 26;19:195. doi: 10.1186/s12879-019-3820-y (PMC6390565; doi:10.1186/s12879-019-3820-y)
Supplement: Supplementary file 6 — Figure S1. Early improvement (ITT population). a. Early improvement at Day 3 in patients with CAP, by risk factor (ITT population). b. Early improvement at Day 4 in patients with HAP (excluding VAP) by risk factor (ITT population). c. Early improvement in high-risk group patients by pathogen type (ITT population). aThe comparator treatment was ceftriaxone ± linezolid in CAP patients and ceftazidime plus linezolid in HAP (excluding VAP) patients. bBetween treatment difference calculated as ceftobiprole minus comparator. cTwo-sided 95% confidence interval is based on a normal approximation to the difference of the two proportions. Early clinical improvement is defined as improved or cured at Day 3 in patients with CAP, and improved or cured at Day 4 in patients with HAP (excluding VAP). Early clinical improvements were evaluated by the investigator, based on an assessment of symptoms using standardised criteria. (PDF 681 kb) [file 12879_2019_3820_MOESM6_ESM.pdf]

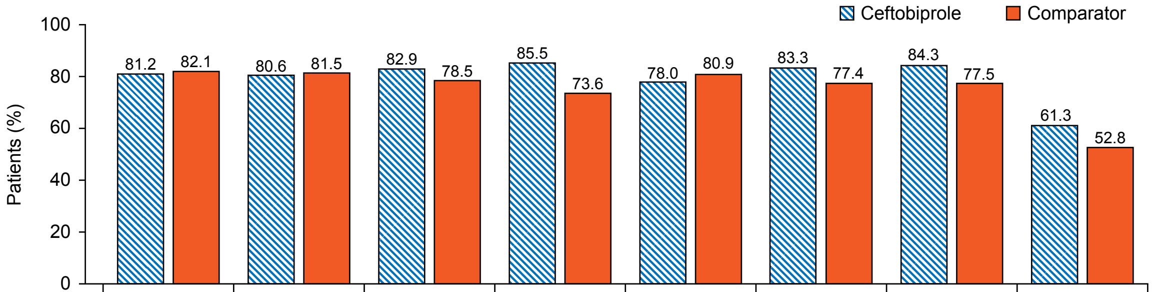

Risk factor	All patients	All high-risk patients	PORT ≥III	PORT ≥IV	Sepsis	Age ≥75 years	COPD	ICU
Number of patients, n (ceftobiprole/comparator[a])	314/324	253/276	158/149	67/72	164/178	54/62	70/80	31/36
Treatment difference (%)[b]	−0.9	−0.9	4.4	11.9	−2.9	5.9	6.8	8.5
95% confidence interval[c]	−6.9, 5.1	−7.6, 5.8	−4.4, 13.2	−1.2, 25.0	−11.4, 5.7	−8.5, 20.3	−5.7, 19.3	−15.2, 32.2

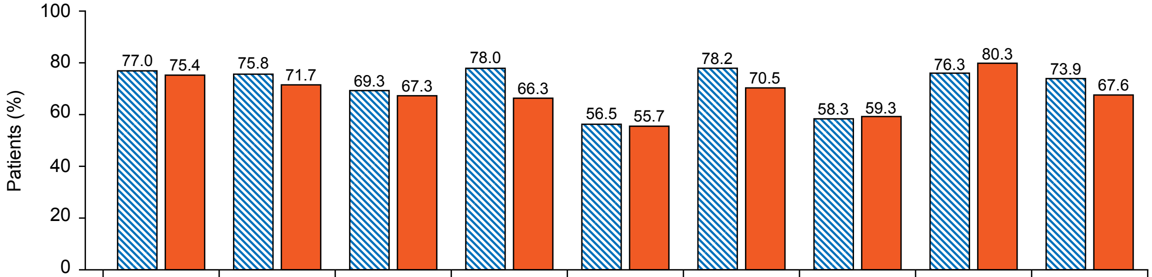

Risk factor	All patients	All high-risk patients	APACHE ≥15	>10 comorbidities	Mechanical ventilation	Age ≥75 years	Bacteremia	COPD	ICU
Number of patients, n (ceftobiprole/comparator[a])	287/284	244/230	101/104	82/92	69/70	78/88	24/27	76/66	111/108
Treatment difference (%)[b]	1.7	4.1	2.0	11.7	0.8	7.8	−0.9	−4.0	6.3
95% confidence interval[c]	−5.3, 8.6	−3.8, 12.0	−10.7, 14.7	−1.4, 24.9	−15.7, 17.3	−5.5, 21.0	−28.0, 26.1	−17.5, 9.6	−5.7, 18.3

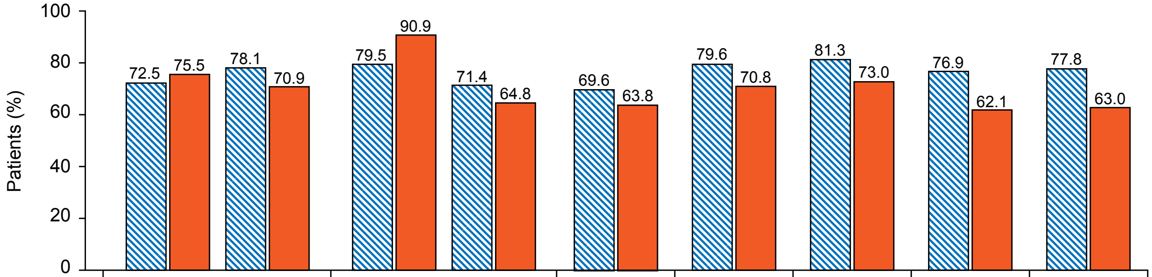

	CAP	HAP (excluding VAP)	CAP	HAP (excluding VAP)	HAP (excluding VAP)	HAP (excluding VAP)	CAP	HAP	HAP
**Pathogen type**	Any Gram-positive		Any Gram-negative		Mixed/ polymicrobial	Any *S. aureus*	Any *S. pneumoniae*	Any MRSA	Any *P. aeruginosa*
Number of patients, n (ceftobiprole/comparator[a])	40/49	73/86	44/44	105/91	46/47	49/65	32/37	26/29	27/27
Treatment difference (%)[b]	−3.0	7.2	−11.4	6.6	5.7	8.8	8.3	14.9	14.8
95% confidence interval[c]	−21.4, 15.3	−6.3, 20.6	−26.0, 3.3	−6.5, 19.7	−13.4, 24.9	−7.0, 24.6	−11.4, 28.0	−9.1, 38.8	−9.2, 38.9

APACHE, Acute Physiology and Chronic Health Evaluation; CAP, community-acquired pneumonia; CI, confidence interval; COPD, chronic obstructive pulmonary disease; HAP, hospital-acquired pneumonia; ICU, intensive care unit; ITT, intention-to-treat; PORT, Patient Outcome Research Team.
