# Supplementary material for: Early improvement in severely ill patients with pneumonia treated with ceftobiprole: a retrospective analysis of two major trials
Source: BMC Infect Dis. 2019 Feb 26;19:195. doi: 10.1186/s12879-019-3820-y (PMC6390565; doi:10.1186/s12879-019-3820-y)
Supplement: Supplementary file 7 — Figure S2. 30-day all-cause mortality (ITT population). a. 30-day all-cause mortality in patients with CAP, by risk factor (ITT population). b. 30-day all-cause mortality in patients with HAP (excluding VAP) by risk factor (ITT population). c. 30-day all-cause mortality in high-risk group patients by pathogen type (ITT population). aThe comparator treatment was ceftriaxone ± linezolid in CAP patients and ceftazidime plus linezolid in HAP (excluding VAP) patients. bBetween treatment difference calculated as ceftobiprole minus comparator. cTwo-sided 95% confidence interval is based on a normal approximation to the difference of the two proportions. (PDF 667 kb) [file 12879_2019_3820_MOESM7_ESM.pdf]

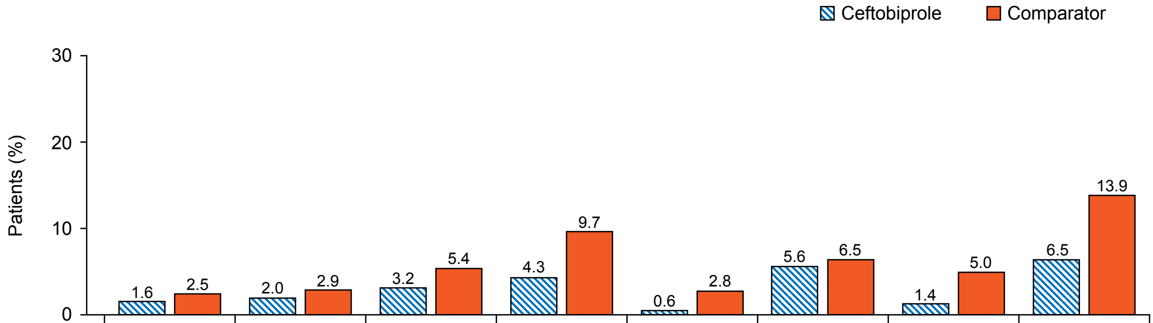

Risk factor	All patients	All high-risk patients	PORT ≥III	PORT ≥IV	Sepsis	Age ≥75 years	COPD	ICU
Number of patients, n (ceftobiprole/comparator[a])	314/324	253/276	158/149	67/72	164/178	54/62	70/80	31/36
Treatment difference (%)[b]	−0.9	−0.9	−2.2	−5.4	−2.2	−0.9	−3.6	−7.4
95% confidence interval[c]	−3.1, 1.3	−3.5, 1.7	−6.7, 2.3	−13.7, 3.0	−4.9, 0.5	−9.5, 7.7	−9.1, 2.0	−21.7, 6.8

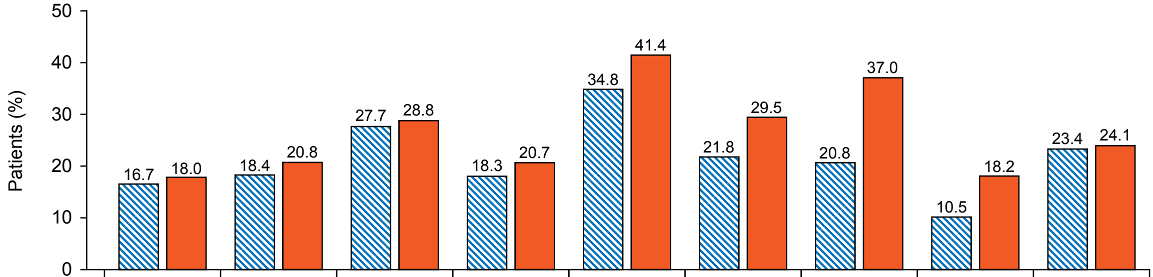

Risk factor	All patients	All high-risk patients	APACHE ≥15	>10 comorbidities	Mechanical ventilation	Age ≥75 years	Bacteremia	COPD	ICU
Number of patients, n (ceftobiprole/comparator[a])	287/284	244/230	101/104	82/92	69/70	78/88	24/27	76/66	111/108
Treatment difference (%)[b]	−1.2	−2.4	−1.1	−2.4	−6.6	−7.8	−16.2	−7.7	−0.7
95% confidence interval[c]	−7.4, 5.0	−9.6, 4.7	−13.5, 11.2	−14.1, 9.4	−22.8, 9.5	−21.0, 5.5	−40.6, 8.2	−19.2, 3.9	−11.9, 10.6

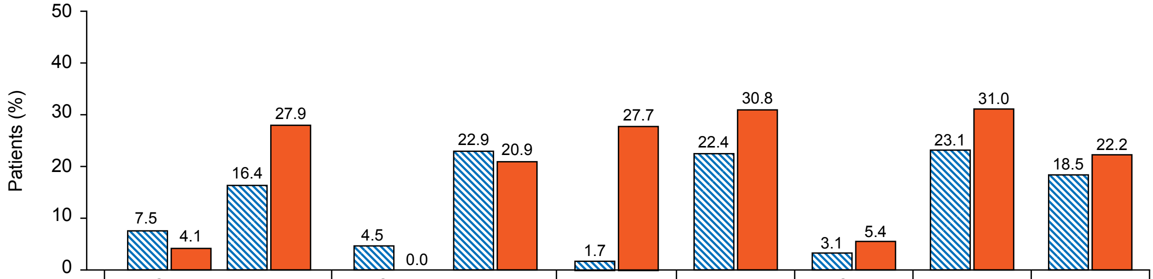

	CAP	HAP (excluding VAP)	CAP	HAP (excluding VAP)	HAP (excluding VAP)	HAP (excluding VAP)	CAP	HAP	HAP
**Pathogen type**	Any Gram-positive		Any Gram-negative		Mixed/ polymicrobial	Any *S. aureus*	Any *S. pneumoniae*	Any MRSA	Any *P. aeruginosa*
Number of patients, n (ceftobiprole/comparator[a])	40/49	73/86	44/44	105/91	46/47	49/65	32/37	26/29	27/27
Treatment difference (%)[b]	3.4	−11.5	4.5	2.0	−5.9	−8.3	−2.3	−8.0	−3.7
95% confidence interval[c]	−6.4, 13.3	−24.2, 1.3	−1.6, 10.7	−9.6, 13.6	−23.4, 11.6	−24.5, 7.9	−11.7, 7.2	−31.3, 15.4	−25.2, 17.8

APACHE, Acute Physiology and Chronic Health Evaluation; CAP, community-acquired pneumonia; CI, confidence interval; COPD, chronic obstructive pulmonary disease; HAP, hospital-acquired pneumonia; ICU, intensive care unit; ITT, intention-to-treat; PORT, Patient Outcome Research Team; VAP, ventilator-associated pneumonia.
